# *Helicobacter pylori* Virulence Factors—Mechanisms of Bacterial Pathogenicity in the Gastric Microenvironment

**DOI:** 10.3390/cells10010027

**Published:** 2020-12-25

**Authors:** Jacek Baj, Alicja Forma, Monika Sitarz, Piero Portincasa, Gabriella Garruti, Danuta Krasowska, Ryszard Maciejewski

**Affiliations:** 1 Department of Anatomy, Medical University of Lublin, 20-400 Lublin, Poland; ryszard.maciejewski@umlub.pl; 2 Chair and Department of Forensic Medicine, Medical University of Lublin, 20-090 Lublin, Poland; aforma@onet.pl; 3 Department of Conservative Dentistry with Endodontics, Medical University of Lublin, 20-090 Lublin, Poland; mksitarz@gmail.com; 4 Clinica Medica “Augusto Murri”, Department of Biomedical Sciences and Human Oncology, University of Bari “Aldo Moro”, 70124 Bari, Italy; piero.portincasa@uniba.it; 5 Section of Endocrinology, Department of Emergency and Organ Transplantations, University of Bari “Aldo Moro” Medical School, Piazza G. Cesare 11, 70124 Bari, Italy; gabriella.garruti@uniba.it; 6 Department of Dermatology, Venerology and Paediatric Dermatology of Medical University of Lublin, 20-081 Lublin, Poland; dana.krasowska@gmail.com

**Keywords:** *Helicobacter pylori*, virulence factor, pathogenicity, urease, cagA, vacA, BabA, SabA, OpiA, DupA

## Abstract

Gastric cancer constitutes one of the most prevalent malignancies in both sexes; it is currently the fourth major cause of cancer-related deaths worldwide. The pathogenesis of gastric cancer is associated with the interaction between genetic and environmental factors, among which infection by *Helicobacter pylori* (*H. pylori*) is of major importance. The invasion, survival, colonization, and stimulation of further inflammation within the gastric mucosa are possible due to several evasive mechanisms induced by the virulence factors that are expressed by the bacterium. The knowledge concerning the mechanisms of *H. pylori* pathogenicity is crucial to ameliorate eradication strategies preventing the possible induction of carcinogenesis. This review highlights the current state of knowledge and the most recent findings regarding *H. pylori* virulence factors and their relationship with gastric premalignant lesions and further carcinogenesis.

## 1. Introduction

*Helicobacter pylori* (*H. pylori*) is a Gram-negative, helix-shaped, microaerophilic, flagellated bacterium that is capable of converting its shape from spiral to coccoid, which is believed to be involved in the enhancement of bacterial survival in the host gastric microenvironment. The spiral form of *H. pylori* enables the successful motility of the bacterium, whereas the coccoid form provides the ability to colonize the mucus layer of the gastric epithelium, enhancing further invasiveness of the bacterium. Furthermore, *H. pylori* is capable of forming biofilms to decrease its susceptibility to several antibiotics, leading to antibiotic resistance mutations and further difficulties with bacterial eradication.

Since 1994, *H. pylori* has been classified as a class I carcinogen associated with the onset of gastric cancer (GC) by the World Health Organization (WHO) as well as by the International Agency for Research on Cancer [1,2]. *H. pylori* colonizes the gastric mucosa of nearly half of the world’s population; however, in the majority of cases, the infected individuals remain asymptomatic. The prevalence of the infections induced by *H. pylori* is estimated at 85–95% in developing countries and approximately 30–50% in developed countries [3,4]. The oral-to-oral and fecal-to-oral routes are two major transmission routes of the bacterium. The adaptation mechanisms of *H. pylori* include the bacterial and environmental factors that enable its survival in the gastric microenvironment where the acidity is at pH even lower than 3.0 [5,6]. The pathogenicity of *H. pylori* is associated with several mechanisms, among which the alterations of the host signaling pathways, indirect inflammatory responses induced within the gastric mucosa, and direct epigenetic outcomes on gastric epithelial cells, are of major importance [7].

The acquisition of *H. pylori* infection occurs most prevalently during childhood, whereas the appearance of gastrointestinal diseases is observed in adulthood [8]. Infection by *H. pylori* significantly contributes to the induction of GC, which is the second most prevalent and the fourth leading cause of death worldwide, leading to more than 740,000 deaths per year [9]. Among patients with GC, *H. pylori* is believed to be the major causation of this malignancy in approximately 92% of patients [10,11]. *H. pylori* infection enhances the progression of the epithelial–mesenchymal transition (EMT) that induces the oncogenic alterations within the gastric mucosa, constituting one of the hallmarks of gastric carcinogenesis [12,13]. Except for GC, *H. pylori* is responsible for the induction and progression of other gastrointestinal impairments and diseases, including gastritis, peptic ulcer disease, dyspepsia, or mucosa-associated lymphoid tissue lymphoma (MALT); contrarily, *H. pylori* is a protective factor against inflammatory bowel disease (IBD) and gastroesophageal reflux disease (GERD) [14,15,16,17,18].

The severity of *H. pylori*-related diseases is associated with numerous virulence factors; a particular genotype of the *H. pylori* strain plays a crucial role. Furthermore, what is of the highest importance is an interplay between the host, gastric microenvironment, as well as bacterial virulence factors. *H. pylori* virulence factors are not only involved in the induction of inflammatory responses, but they also control and regulate those responses, maintaining chronic inflammation. *H. pylori* virulence factors enable the colonization and survival of the bacterium within the gastric mucosa, leading to further immune escape and ultimately, the induction of premalignant alterations. *H. pylori* exhibits an expanded complex of mechanisms that alters host cellular responses and signaling pathways (Table 1).

## 2. Urease

Urease is one of the most essential *H. pylori* virulence factors involved in bacterial metabolism and colonization within the gastric mucosa; it is the most abundantly expressed protein by this bacterium. *H. pylori* urease can be found in both—the bacterial cytoplasmic compartment and on the surface of the bacteria; thus, two types of urease can be distinguished based on its localization—internal and external [19]. External urease is primarily produced during the cell lysis and works most sufficiently at pH between 5.0 and 8.5, whereas the internal urease shows its activity at pH between 2.5 and 6.5. The enzymatic reaction of urease is based on the hydrolysis of urea into ammonia and carbamate, which is further decomposed into another molecule of ammonia and carbonic acid that eventually induces the increase in gastric pH; the whole process is nickel-dependent. Paradoxically, *H. pylori* colonization might be terminated when the mucus pH will rise above 8 pH; this mechanism is probably induced in order to prevent the excessively high amounts of bacteria in the gastric mucus [20]. Except for urease, *H. pylori* induces the production of other ammonia-producing enzymes such as aliphatic amidases E and F (AmiE and AmiF) [21].

Urease is a critical factor that facilitates bacterial colonization within the gastric mucosa; urease-negative mutants fail in colonizing the gastric mucosa at physiological pH levels as sufficiently as urease-positive *H. pylori* strains [22]. Except for facilitated colonization of *H. pylori*, urease protects the bacterium from gastric acidity due to the increased release of ammonia, promotes bacterial nutrition via the release of the host metabolites, and also generates the proton motive force during the hydrolysis of urea [22]. Furthermore, urease modulates host immune responses via several mechanisms, including altered opsonization, enhanced chemotaxis of neutrophils and monocytes, facilitated apoptosis due to binding to the class II major histocompatibility complex (MHC) receptors, or enhanced release of the pro-inflammatory cytokines [23]. Urease also takes part in platelet activation via the lipoxygenase-derived eicosanoids [24]. Urease is believed to stimulate angiogenesis, leading to the enhanced progression of GC [25]. In *H. pylori* species, urease activity is controlled by both the environmental pH and the availability of the cofactor nickel; bacterial cultures that are supplemented with nickel tend to exhibit significantly increased urease activity [26]. *H. pylori* in the spiral form presents higher urease activity compared with the bacterium present in the coccoid form [21]. Moreover, urease and catalase activity, as well as protein synthesis by *H. pylori*, might be impaired at very low pH [27]. Greater urease activity might be associated with a higher risk of induction of the histopathological alterations within the gastric mucosa and further gastric carcinogenesis [28]. Urease activity might be modulated by the *flbA* gene, the same gene that is involved in flagellar biosynthesis [29]. The inhibition of urease is considered to constitute a potential therapeutic strategy preventing *H. pylori*-related diseases [30].

## 3. Flagellum

The *H. pylori* flagellum constitutes a crucial factor enabling bacterial motility and chemotaxis properties. Physiologically, *H. pylori* possesses a bundle of 2–6 sheathed unipolar flagella; each of the flagella is approximately 3 μm long and the surrounding sheaths enable protection from the gastric acidic microenvironment [31,32]. The speed of *H. pylori* movement is associated with the exact number of flagella and might differ among species [33]. Two flagellin proteins—FlaA and FlaB—constitute major components of the flagellar filament. It was observed that FlaA-negative *H. pylori* mutants do not produce flagella at all, whereas FlaB-negative mutants might produce flagella; however, they are not fully functional [34]. Kao et al. (2014) demonstrated that *csrA H. pylori* mutants showed decreased motility and adherence properties compared to the wild-type *H. pylori* presenting the relevance of *csrA* in the flagellar formation [35]. The motility of the flagella is provided by the proton motive force, which might explain bacterial chemotactic movements towards urea as urease-driven hydrolysis provides the generation of the proton motive force specifically [36,37].

Flagellum, as a factor that provides bacterial motility, enables further colonization in the gastric mucosa; therefore, it constitutes one of the most crucial virulence factors associated with *H. pylori* pathogenicity specifically at the very first stages of bacterial invasion. It was demonstrated that less motile strains of *H. pylori* present difficulties with further colonization within the gastric mucosa; furthermore, bacterial motility correlates with bacterial infectivity as well [38]. It was observed that non-flagellated *H. pylori* mutants are not capable of colonizing the gastric mucosa, providing an insight that bacterial motility is fundamental for further bacterial pathogenicity [39]. Flagellar motility is controlled by the so-called chemotaxis pathway that controls bacterial motility and its relationships with the microenvironment [40,41]. Except for colonization, flagella are believed to be involved in *H. pylori*-induced inflammation and immune invasion; furthermore, flagella take part in biofilm formation [42,43]. Several flagellins that compose bacterial flagella such as HpA, FlaA, or FlaB might promote humoral immunity and stimulate specific antibody responses after infection [44,45]. It was observed that *H. pylori* strains that possess highly motile properties induce an increased release of IL-8 [46,47]. It was suggested that genes that regulate flagellar synthesis might also be involved in the regulation of other virulence factors such as adhesins [48].

## 4. Cytotoxin-Associated Gene A

*H. pylori* strains can be generally categorized into two subtypes—cytotoxin-associated gene A (CagA)-positive and CagA-negative *H. pylori* strains. CagA along with a type 4 secretion system (T4SS) are encoded by the cag pathogenicity island (cagPAI) that plays a key role in carcinogenesis. CagA is injected into the cell via the pilus formed by T4SS and induces cellular alterations that impair cell motility, cellular proliferation, and apoptosis, as well as alters the arrangement of the whole cytoskeleton. The adherence of *H. pylori* to the gastric epithelial cells induces the expression of cagA and the process seems to be controlled by the Fur protein among others [49]. The presence of CagA is usually associated with a higher prevalence of inflammatory responses and greater damage induced within the gastric mucosa; however, the CagA status alone is not the only decisive factor regarding further gastrointestinal impairments induced by *H. pylori*.

Generally, infections by CagA-positive *H. pylori* strains are associated with greater severity and worse clinical outcome of patients. Kyrillos et al. (2015) showed that *cagA* expression might be associated with the bacteriophage genes (phage orthologous genes) affecting *H. pylori* virulence [50]. CagA-positive *H. pylori* strains stimulate the elevation of IL-8 and IL-12 levels in the sera of infected individuals [51,52]. The amounts of interleukins that are secreted during *H. pylori* infection are highly associated with the number and the variations within C-terminal Glu-Pro-Ile-Tyr-Ala (EPIYA) motifs; for instance, *H. pylori* strains with the EPIYA-D motif are prone to release higher amounts of IL-8 compared to other variations [53]. An animal model also showed that CagA-positive strains with variations in the EPIYA C motifs might affect the number of gastrin-producing G cells and somatostatin-producing D cells [54]. Lai et al. (2008) observed that cholesterol might inhibit excessive CagA-related IL-8 release as well as the “hummingbird” phenotype [55].

CagA-positive strains are more motile compared to the CagA-negative strains, which indicates that CagA is also associated with bacterial motility [56]. Furthermore, an upregulation of CagA in gastric cells requires the flagellar regulatory system of *H. pylori*, indicating the cooperation between *H. pylori* motility and CagA presence [57]. CagA promotes carcinogenesis via numerous pathways affecting the activity of tumor suppressor proteins such as run-related transcription factor 3 (RUNX3) or apoptosis-stimulating protein of p53 2 (ASPP2) [58]. *P53* mutations are more prevalent in tumors induced by CagA-positive strains than the negative ones [59]. *CagA* gene presents several variants and there is a correlation between the particular cagA genotype and patients’ ethnicity [60,61,62,63,64,65,66,67]. CagA presents antiapoptotic effects, stimulates motility, and induces host cell growth with proliferation. Choi et al. (2019) showed that enhanced gastric carcinogenesis might be associated with the overexpression of the transcription factor CDX1, which is stimulated by the CagA-positive strains [68]. Moreover, CagA is highly associated with the induction and further progression of the EMT within the gastric mucosa, which is associated with greater invasiveness properties. The process is stimulated via several CagA-related mechanisms such as the downregulation of programmed cell death protein 4 (PDCD4), induction of cancer stem cell-like properties, reduction in glycogen synthase kinase 3 (GSK-3) activity, overactivation of fibroblasts, suppression of microRNA-134, altering the yes-associated protein (YAP) pathway, or Afadin protein downregulation [69,70,71,72,73,74,75,76]. Particular *cagA* genotypes present different severity of enhanced IL-8 and cytotoxin production, inflammatory responses, or apoptosis in gastric epithelial cells. CagA represses host heat shock proteins’ expression by downregulating the levels of host HSPH1 (HSP105), HSPA1A (HSP72), and HSPD1 (HSP60) [77].

Infection by the CagA-positive strains promotes the induction of gastritis (active or atrophic), duodenal ulcers, and gastric carcinogenesis; [57,78,79]. The co-expression of CagA, outer inflammatory protein A (OipA), vacuolating cytotoxin (VacA), and blood group antigen-binding adhesin (BabA) significantly enhances the inflammatory responses, ultimately worsening the clinical outcome of patients and increasing the probability of greater gastrointestinal diseases incidence [80]. Even though CagA-positive strains seem to be much more pathogenic than the CagA-negative strains, CagA-positive strains are easier to eradicate, according to the meta-analysis by Wang et al. (2017) [81].

## 5. Vacuolating Cytotoxin A

Vacuolating cytotoxin A (VacA) is a cytotoxin involved in the formation of pores where mechanisms of action and final pathogenicity slightly differ depending on the time of exposure to the host cells. In cases of acute exposure, VacA promotes the autophagy pathways in cells, whereas, during chronic exposure, VacA promotes the appearance of impaired autophagosomes as well as induces the formation of intracellular vacuoles that enable the survival of *H. pylori* in the host cells [82]. VacA constitutes one of the most crucial virulence factors that enable bacterial colonization and survival in the gastric epithelium; the *vacA* gene is present in all of the *H. pylori* strains. Receptor-like protein tyrosine phosphatases α and β (RPTP—RPTPα and RPTPβ), low-density lipoprotein receptor-related protein-1 (LRP-1), or sphingomyelin are major types of receptors to which VacA binds [83,84]. So far, several *vacA* genotypes are distinguished including s1, s2, m1, m2, s1m1, s1m2, s2m2, and s2m1. The VacA s1 genotype is one of the most abundant in *H. pylori*-infected patients and there is an association between this genotype and peptic ulcer disease [85]. VacA s1m1 is commonly found in *H. pylori*-infected patients with chronic gastritis, whereas in *H. pylor*i-induced gastric cancer, usually s1 and m1 VacA genotypes are most prevalent [86,87]. It was suggested that VacA polymorphism might be associated with the clinical phenotype of the *H. pylori* infection.

Except for autophagy and disruption of the endosomal and lysosomal functions, VacA is involved in many other processes that impair gastric epithelial cells such as alterations in mitochondrial functioning, as well as apoptosis and necrosis [88]. Furthermore, VacA significantly affects the immune cells by inhibiting the activation and proliferation of T cells and B cells and inducing apoptosis in macrophages primarily by inhibition of the IFN-β signaling; VacA is also involved in the induction of an excessive release of IL-8 [89,90]. Furthermore, VacA stimulates the differentiation of the regulatory T cells into effector T cells, which also contributes to the persistence of *H. pylori* infection [91]. Gastric epithelial cells are targeted by the VacA-containing endosomes that afterward activate several cellular degradative systems inducing host immune-inflammatory responses and further damage of the organelles [92].

Abdullah et al. (2019) demonstrated a synergy between VacA and CagA, showing that CagA might accumulate within the VacA-induced impaired autophagosomes [93]. This synergy is also observed in cases of the acquisition of specific nutrients (such as iron) by the bacterium, wherein VacA and CagA interplay with one another. VacA inhibits the Erk1/2 kinase pathways preventing cellular elongation induced by CagA [94]. Moreover, there is evidence of synergy between VacA, CagA, and BabA; an interplay between those factors significantly worsens the inflammatory responses and is associated with a higher incidence of intestinal metaplasia [80]. It was demonstrated that weak bases such as ammonia released in excessive amounts during *H. pylori* infection might significantly stimulate cell death via the enhancement of VacA activity [95]. Similar to CagA, VacA is also associated with phage orthologous genes, enhancing *H. pylori* pathogenicity [50].

Various *vacA* genotypes are further associated with different morbidity incidents of such gastrointestinal disorders as GC or peptic ulcer disease, as well as the clinical outcome of patients [66,78,96,97,98,99,100,101,102]. VacA-positive strains are associated with a greater prevalence of gastric premalignant lesions, as well as GC [103]. Different *vacA* genotypes might also be associated with the severity of *H. pylori*-induced inflammation [104]. Furthermore, particular *vacA* genotypes might induce the enhanced expression of the transforming growth factor-beta 1 (TGF-β1) mRNA levels [105]. Similar to *cagA*, *vacA* expression might be stimulated in a Fur-dependent manner [49]. Figura et al. (2015) demonstrated that *vacA* polymorphism might correlate with the histological type of GC; this characteristic is not applicable in the case of *cagA* [106]. It was proven that *vacA* s1 strains are far easier to eradicate compared to *vacA* s2 strains [81]. However, VacA is involved in the formation of intracellular reservoirs enabling *H. pylori* survival in the gastric environment even after eradication therapy [107].

## 6. Catalase

Catalase is an enzyme that converts hydrogen peroxide (H_2_O_2_) into water (H_2_O) and molecular oxygen (O_2_) and is one of the most abundant proteins in both—plant and animal cells. Three unrelated types of catalase can be distinguished and those include the “classical” catalase, catalase-peroxidase, and manganese-containing catalase. Catalase is implicated in numerous pathological processes such as inflammation, apoptosis prevention, as well as induction of mutagenesis resulting in a vast number of tumors. Catalase presents its activity in the cytoplasm and periplasm; it can also be surface-expressed [108].

Catalase levels are estimated at 4–5% of *H. pylori* total protein content and it is one of the most highly expressed proteins in *H. pylori* strains obtained from the gastric mucosa [109,110]. *H. pylori* catalase is more resistant to inhibition by cyanide or aminotriazole, compared to catalase from other species [111]. It was demonstrated that catalase is involved in bacterial protection from oxidants and oxidative stress, suggesting its heterogeneous ways of action during infection [112]. Furthermore, Richter et al. (2016) showed that catalase might be involved in the protection of *H. pylori* from complement-mediated killing and this process is strongly associated with the vitronectin (Vn) that regulates the complement system [113]. The authors also showed that catalase, as a Vn-binding protein, might enhance the evasion of an infected individual’s immunity. Catalase activity is also crucial in the maintenance of bacterial survival at the cell surface of the phagocytes [114]. Catalase also enhances *H. pylori* survival within the macrophage phagosomes [115]. An animal model showed that mice lacking catalase showed significantly decreased ability to colonize in the gastric mucosa, suggesting a role of catalase in *H. pylori* colonization [116]. *H. pylori* catalase prevents the bacterium from toxic long-chain fatty acids and their metabolites as well as provides *H. pylori* protection from phagocytosis; catalase is also involved in the modulation of immune responses [117,118,119]. *H. pylori* catalase activity is controlled by Fur protein and iron concentrations; it was demonstrated that strains of *H. pylori* that were Fur-deficient and grown on a medium with low iron concentrations showed significantly reduced catalase activity [120]. It was demonstrated that a vaccination that contains pcDNA3.1 encoding *H. pylori* catalase suppresses further bacterial colonization and inflammatory responses induced in gastric mucosa; furthermore, humoral immune responses are induced by such a vaccination [121].

## 7. Superoxidase Dismutase

Superoxide dismutase (SOD) is one of the *H. pylori* enzymes that prevents the bacterium from reactive oxygen species (ROS), enabling the maintenance of the proper homeostasis. Contrarily to catalase, SOD is located on the cell surface only. SOD facilitates the dismutation of superoxide to oxygen, preventing excessive amounts of toxic superoxide free radicals. SOD, which is a metalloenzyme, can be categorized into three isoforms depending on its properties and chemical composition—copper-zinc SOD (Cu/Zn-SOD), manganese SOD (Mn-SOD), and extracellular matrix SOD (EC-SOD). *H. pylori* SOD requires iron (Fe) for proper and effective functioning, acting as a SOD cofactor. Any alterations in the cellular Fe concentrations decrease SOD activity, enhancing cellular susceptibility to oxidative damage.

*H. pylori*-infected individuals with moderate to severe gastric mucosa inflammation present greater expression of Mn-SOD than individuals with the intact gastric mucosa. Mn-SOD expression was observed to be more enhanced primarily within the pit cells of the antrum and not the corpus [122]. It was reported that exposure to the CagA-positive *H. pylori* strains results in significantly greater SOD, catalase, and glutathione peroxidase activities compared to exposure to the CagA-negative strains [123]. SOD is involved in the inhibition of the HOCl and NO/peroxynitrite signaling pathways, enhancing the survival of the already transformed cells from apoptosis [124]. Negovan et al. (2018) demonstrated that MnSOD Ala16Val polymorphism might be associated with a higher risk of reactive gastropathy [125]. *H. pylori* SOD inhibits the production of the pro-inflammatory cytokines (by downregulating the nuclear factor kappa B (NF-κB) activation pathways), as well as macrophage inflammatory protein 2 (MIP-2) (IL-8 homolog) via activation of the macrophages [126]. Noguchi et al. (2002) demonstrated that only Mn-SOD and not Cu/Zn-SOD provides effective protection against ROS [127]. SOD-deficient *H. pylori* mutants present a greater susceptibility to oxidative damage as well as fail in colonizing the gastric mucosa, presenting the relevance of SOD in the proper bacterial colonization process. Except for colonization, SOD expression is crucial for bacterial growth and survival primarily in the host microenvironment with a high intensity of oxidative stress [128]. Furthermore, there is a relationship between SOD activity and the onset and progress of gastric diseases [129]. 

## 8. Lewis Antigens

Lewis (Le) antigens are fucosylated glycolipids that constitute one of the components of the *H. pylori* O-specific chain of the lipopolysaccharide (LPS) and the majority of *H. pylori* strains express at least one type of Le antigen. Le antigens are expressed on the gastric epithelial cells; thus, *H. pylori* molecular mimicry in the form of Le antigens expression on the bacterial cell surface provides one of the most effective mechanisms enabling *H. pylori* colonization within the gastric mucosa, evoking the host immune responses. The expression of Le antigens on the bacterial cell surface prevents the bacterium from the host defense mechanisms promoting bacterial survival. Le antigens expression within the gastric epithelial cells might differ depending on the clinicopathological features of the infected patients and different Le types might be expressed depending on whether a patient will develop chronic gastritis or GC specifically [130]. Except for colonization, Le antigens are also crucial for the bacterial adherence to the cells and further internalization that induces chronic and persistent *H. pylori* infection [131,132].

Gastric epithelial cells and LPS might express various Le antigens at the same time, among which two major Le antigen types can be distinguished—type 1 (Le^a^ and Le^b^) and type 2 (Le^x^ and Le^y^). It is estimated that approximately 80–90% of *H. pylori* strains express Le^x^ or Le^y^ [133]. Le antigens’ expression patterns might differ depending on the particular area in gastric mucosa as well as the intensification of inflammation or metaplastic alterations. Moreover, Le antigens’ expression is associated with the particular geographic origin of the *H. pylori* strain [134]. Le^b^ expressed on the gastric epithelial cells constitutes the major site of the *H. pylori* attachment; however, there are incidents of bacterial colonization that are independent of particular Le antigen expression [135,136]. *H. pylori* Le antigens expression depends on the time of the bacterial growth being mostly expressed in a growth phase-dependent manner. Different patterns of Le antigens’ expression were observed in adults and children—for instance, Le^b^ expression correlates with the bacterial distribution within the antrum and corpus of the adult patients, whereas in children, it might increase the risk of ulceration [137,138]. Furthermore, there are differences in the Le antigens’ expression regarding symptomatic and asymptomatic *H. pylori*-infected individuals; asymptomatic individuals tend to present significantly lowered expression of Le^x^ antigen and the complete absence of type 1 Lewis antigens, compared to the infected symptomatic individuals [139].

Martin et al. (2006) demonstrated a relationship between the ABO group and Le antigens expression, indicating that individuals with 0 Le (a-b+) and A_2_ Le (a-b+) phenotypes might be more susceptible to *H. pylori* infection [140]. Anti-Le antibodies are present in the sera of patients infected by *H. pylori* as well as those with an *H. pylori*-induced GC and according to some research, this response is strictly associated with the bacterial Le phenotype [141]. Furthermore, the presence of the Le^x^ IgM antibodies in the gastric lumen enhances the adhesive properties of *H. pylori* strains, primarily those which express high amounts of the Le^x^ antigen [142]. However, it was demonstrated that outer membrane vesicles (OMVs), produced during *H. pylori* invasion, have the ability to absorb anti-Le antibodies from the sera of infected individuals, enabling further *H. pylori* growth and colonization [143,144].

## 9. Arginase

Arginases are Mn^2+^-metalloenzymes belonging to the ureohydrolase family that catalyze the hydrolysis of L-arginine into L-ornithine and urea during the urea cycle [145]. There are two distinct isoforms of arginase—type I and II—that have been identified in eukaryotes. Arginase I is mainly expressed in the liver and is involved in the last step of the urea cycle, whereas arginase II is a mitochondrial enzyme responsible for L-arginine homeostasis. Arginases are the antagonists of the inducible nitric oxide synthase (iNOS); they also stimulate apoptosis and prevent bacterial killing.

*H. pylori* arginase is encoded by the *RocF* gene; arginase is crucial for bacterial colonization since it provides acid resistance in the gastric microenvironment. *H. pylori* arginase shows a similarity to arginase II, however, with the presence of additional acidic residues near the N-terminus. Contrarily to other arginases, *H. pylori* arginase exhibits higher catalytic activity with Co^2+^ or Ni^2+^ rather than Mn^2+^ as a cofactor and shows greater activity at the acidic pH instead of the alkaline; the optimal activity of arginase is shown at pH 6.1 [146,147]. The animal studies have shown that arginase-deficient *H. pylori* are more prone to be killed by the macrophages in a NO-dependent manner; arginase also inhibits the proliferation of the T cells mainly by the reduction in CD3ζ expression [148]. Furthermore, arginase inhibits the production of NO which eventually impairs the bactericidal activity of the macrophages [149]. The major mechanism of arginase-associated impairments of the host immune responses is due to the downregulation of NO production [150]. It was demonstrated that during the acute stages of *H. pylori* infection, the inhibition of arginase increased NO and iNOS production in the macrophages [151]. *H. pylori* arginase expression alters the immune responses by affecting M1 macrophage activation, diminishing Th1/Th17 T-cell differentiation, as well as controlling the IL-8 transcription in gastric epithelial cells [152,153]. Moreover, the upregulated expression of arginase II in *H. pylori*-infected tissues induces the apoptosis of the macrophages [154]. Arginase is essential for *H. pylori* survival in the acidic microenvironment; however, it is not crucial for bacterial colonization. 

## 10. Phospholipases

Outer membrane phospholipase A (OMPLA) of *H. pylori* is involved in the degradation of various lipids, enabling effective bacterial permeability, especially during stress conditions. Furthermore, *H. pylori* synthesizes phospholipase A2 (PLA_2_), phospholipase A1 (PLA_1_), as well as phospholipase C (PLC) and phospholipase D (PLD) that degrade phosphatidylcholine and phosphatidylethanolamine [155]. Due to the damages of the mucus layer induced by the *H. pylori* phospholipases, gastric epithelial cells become progressively impaired and lose their physiological functions. Except for mucosal damage, phospholipases promote the chronic inflammation that might further induce the formation of peptic ulcers. This enables further bacterial colonization and survival within the gastric microenvironment. It was demonstrated that *H. pylori* strains that secrete greater amounts of phospholipases significantly increase the risk of chronic gastritis and subsequent carcinogenesis [156]. The secretion of phospholipases is also associated with the induction of several inflammatory responses by the stimulation of arachidonic acid (a precursor of prostaglandins and leukotrienes) and lysophospholipids production. Sitaraman et al. (2012) showed that *H. pylori* PLD activates the ERK1/2 signaling pathway in gastric epithelial cells, inducing further cellular impairments [157]. 

## 11. Lipopolysaccharide

Lipopolysaccharide (LPS) is the component of the *H. pylori* outer membrane that constitutes one of the most essential virulence factors, enabling the induction and progression of a chronic bacterial infection. LPS is composed of three major parts, including the core oligosaccharide, the lipid-A region, and the O-antigen; cholesterol is considered to play a crucial role in the maintenance of the LPS structure [158,159]. It was demonstrated that O-antigen can directly induce the host inflammatory responses due to molecular mimicry [160,161,162,163,164]. Iron availability is crucial for LPS expression by *H. pylori*; it was observed that iron-deficient strains expressed significantly lower amounts of LPS [165]. One of the intermediate metabolites of LPS, heptose-1,7-bisphosphate (HBP), plays a role in the induction of the pro-inflammatory responses as well as IL-8 release in human gastric epithelial cells [166]. Furthermore, a release of HBP by LPS translocation via T4SS facilitates NFκB-dependent inflammation [167,168].

LPS was reported to differ among particular *H. pylori* strains primarily by glucan-heptane linker presence or absence, depending on the geographical occurrence of the *H. pylori* strains [169]. Being embedded in the bacterial outer membrane, LPS constitutes a barrier protecting *H. pylori* from the compounds that could be potentially toxic for the bacterium. Yokota et al. (2012) proposed the division of LPS into two antigenic phenotypes—weakly antigenic epitope-carrying LPS and highly antigenic epitope-carrying LPS that is most prevalently expressed in GC patients with chronic and severe inflammation [170].

*H. pylori* LPS is recognized by toll-like receptor 4 (TLR4)/MD-2 where toxic activity is slightly slower and weaker compared to other Gram-negative bacteria species. Some research demonstrated that several *H. pylori* strains might act as TLR4 antagonists [171]. Apart from TLR4, LPS activates TLR-2 expressed in the cellular membrane, inducing the NFκB-dependent expression of the -4, -6, -7, and -9 claudins as well as enhanced Th1 immune responses [172,173]. The expression of the above-mentioned claudins in GC patients is associated with poorer clinical outcome and shorter survival. It was demonstrated that gastric carcinogenesis might be promoted via LPS-TLR4 pathways during *H. pylori* infection [174]. LPS type 1 (but not type 2) has an ability to activate TAK1 and TAB1, inducing the enhanced expression of mitogen oxidase 1 (Mox1) and p67-*phox* also via the stimulation of TLR4 in gastric pit cells [175]. Moreover, NFκB activation via LPS stimulates overexpression of the programmed death-ligand 1 (PD-1) that significantly contributes to the progression of gastric carcinogenesis [176].

LPS enables chronic *H. pylori* persistence that significantly contributes to the induction of the host inflammatory responses, resulting in chronic inflammation, peptic ulcers, gastritis, and further gastric carcinogenesis. Furthermore, LPS presents a capacity to activate neutrophils, enhancing the onset of the oxidative stress reactions [177]. Gastric inflammation and cellular proliferation might be stimulated by the MEK1/2-ERK1/2 mitogen-activated protein kinase cascade that is regulated by LPS [178]. LPS can form an LPS-TLR4-myeloid differential protein-2 (MD-2) multimer that promotes further enhanced cytokine release and enhanced activation of the pro-inflammatory signaling pathways. Furthermore, the TLR4-MD-2 pathway significantly affects GC proliferation and migration and the whole process is induced by the LPS-mediated overexpression of CXC chemokine receptor 7 (CXCR7) [179]. Inflammatory responses within the gastric microenvironment can also be enhanced by the LPS-related protein kinase Cδ (PKCδ)-mediated phosphorylation of spleen tyrosine kinase (Syk), pathways activated after LPS binding to TLR4, MMP-9 secretion, as well as PLC/PKC/PI3K activation [180,181]. LPS is involved in the induction of monocyte inflammatory responses as well as monocyte transendothelial migration; it was demonstrated that LPS might be a major activating factor of several monocyte functions [182]. LPS mediates IL-8 and IL-1β release from the macrophages, whereas *IL-1β* gene polymorphism is associated with the risk and severity of *H. pylori*-induced GC [183,184]. Furthermore, LPS mediates the release of excessive amounts of other interleukins and immune mediators such as TNF-α, IFN-γ, IL-10, IL-12, IL-18, epithelial neutrophil-activating peptide 78 (EPA-78), and monocyte chemotactic protein 1 (MCP-1) [185,186,187]. Pan et al. (2013) demonstrated that there is a relationship between T309G polymorphism in the *MDM2* gene, *H. pylori* infection, and further gastric carcinogenesis, and the whole process might be intensified by LPS [188]. LPS regulates the expression of Pellino (1 and 2) proteins that stimulate further pro-inflammatory chemokine release, triggering inflammation [189]. LPS dysregulates the secretion of mucin, contributing to the disruption of the gastric mucosal mucus via the overactivation of caspase-3 and further release of proapoptotic factors; the process is regulated by ERK and p38 kinase [190]. LPS also contributes to the secretion of excessive amounts of pepsinogen [191]. Another virulence mechanism of LPS includes the capability to stimulate bacterial adherence and colonization within the gastric mucosa via LPS interaction with the trefoil factor family (TFF) protein TFF1 [192]. LPS is involved in the activation of the JAK/STAT pathways significantly promoting gastric carcinogenesis; the mechanisms include direct LPS-related phosphorylation of JAK1, JAK2, and STAT3 or indirect upregulation of JAK and STAT members via miR-375 and miR-106b inhibition [193].

## 12. Blood Group Antigen-Binding Adhesin

Blood group antigen-binding adhesin (BabA) is a virulence factor that enables *H. pylori* adherence to the gastric epithelium and delivery of toxins (such as CagA or VacA) or other virulence factors into the host cells, promoting either direct or indirect damage of host tissues via inflammatory or immune responses. It was proposed that BabA expression in gastric tissue samples might constitute a biomarker of *H. pylori* infection [194]. The expression of the *babA* gene is strongly associated with the presence of *cagA* in particular *H. pylori* strains; it also depends on the particular geographic area where specific strains are more or less prevalent [195,196]. Furthermore, the co-expression of *babA*, c*agA*, and *vacA* might contribute to the onset and progression of paraneoplastic gastric lesions as well as other gastrointestinal disorders. BabA, CagA, and VacA present a synergy, worsening the severity and clinical outcome of the gastritis patients [197]. Apart from bacterial adherence and colonization, BabA stimulates several immune responses such as granulocyte infiltration or a release of IL-8 enhancing gastric inflammation [198]. Expression of BabA by *H. pylori* strains is significantly associated with the onset, progression, as well as severity, and clinical outcome of several gastrointestinal diseases such as peptic ulcer disease or GC [199]. BabA expression also correlates with greater mucosal inflammation.

BabA adheres to H-type 1 and ABO/Lewis b (Le^b^) blood group antigens expressed in the gastric epithelium facilitating TSS4 activity, releasing excessive amounts of the pro-inflammatory factors stimulating carcinogenesis [200,201,202,203]. Furthermore, *H. pylori* binding to MUC5AC mucin in the gastric epithelium is also BabA-dependent [204]. BabA-stimulated increased TSS4 activity is associated with more severe inflammation, development of intestinal metaplasia, and malignant transformations [205]. BabA is primarily detected in *H. pylori* strains obtained from symptomatic patients (with GC or duodenal ulcer disease). BabA is capable of binding to its receptors both in the mucosa of the oral cavity and gastric mucosa. It was demonstrated that there is a relationship between BabA expression and the severity of gastritis in the gastric antrum [206]. Saberi et al. (2016) showed that *H. pylori* strains with low BabA expression and decreased capability of binding to the Le^b^ can be detached from the gastric mucus and stimulate ulceration within the duodenum, increasing the risk of peptic ulcer disease [207]. Moreover, low BabA levels are more likely to induce more severe and chronic gastrointestinal diseases, compared to strains with high BabA expression. BabA-expressing *H. pylori* strains are capable of inducing histological alterations within the gastric mucosa and stimulate the infiltration of inflammatory mediators; there are no differences between BabA-positive and -negative strains in terms of colonization properties [208]. The co-expression of *babA* and *cagA* genes, as well as bacterial coccoid form, significantly affect the severity of gastritis [209]. Furthermore, BabA expression is more prevalent in CagA-positive *H. pylori* strains compared to CagA-negative strains [210]. BabA along with sialic acid-binding adhesion (SabA) enables *H. pylori* adherence to the spasmolytic polypeptide-expressing metaplasia (SPEM) glands, promoting metaplastic alterations that contribute to the onset of carcinogenesis [211].

## 13. Sialic Acid-Binding Adhesin

Sialic acid-binding adhesin (SabA) is an adhesin belonging to the outer membrane protein (OMP) family that facilitates *H. pylori* adherence to gastric epithelial cells via binding to the Le^x^ antigen. It is estimated that SabA is present in approximately 40% of *H. pylori* strains. SabA expression is significantly increased during bacterium-induced inflammation within the gastric microenvironment. Enhanced expression of OMPs including SabA, BabA, and OipA is associated with the modulation of gastrointestinal disorders. The expression of SabA is associated with the progression of gastric diseases, excessive neutrophil infiltration, and gastric atrophy during infection; furthermore, there is a relationship between SabA expression and the extent of bacterial colonization [212,213]. There is a relationship between SabA expression and severe intestinal metaplasia, gastric atrophy, as well as further gastric carcinogenesis [214]. Except for the stimulation of neutrophil infiltration, SabA presents a capability to activate human neutrophils as well (through the selectin mimicry), contributing to persistent inflammation via the induction of oxidative damage [215]. Furthermore, SabA can be stimulated by *H. pylori* neutrophil-activating protein (NAP) [216]. Similarly to BabA, SabA, along with OipA, are considered to improve diagnostic accuracy; thus, they could potentially act as diagnostic biomarkers of active *H. pylori* infection and *H. pylori*-induced GC [217]. The enhanced expression of the *saba* gene was demonstrated to be one of the triggers that induce an iron deficiency in *H. pylori*-infected patients [218]. Furthermore, it was shown that iron deficiency might further promote the overexpression of *saba*. Genetic diversity of the *saba* gene depends on particular geographical origin; moreover, a particular genotype might influence the clinical outcome of *H. pylori*-infected patients [219].

## 14. Outer Inflammatory Protein A

Outer inflammatory protein A (OipA), encoded by the *hopH* gene, belongs to the OMPs family and as an *H. pylori* virulence factor, it is strongly associated with the bacterial adherence, colonization, induction, and progression of gastrointestinal disorders, and might affect the clinical outcome of the infected patients [220]. OipA-positive *H. pylori* strains are more prone to induce much more severe inflammation in gastric mucosa compared to OipA-negative strains. Binding of the OipA to the gastric epithelial cells activates the apoptotic cascade in the host cells primarily via the Bcl-2 family pathway and increased levels of Bax and intracellular cleaved-caspase 3 [221]. Damage induced by OipA presence is usually correlated with a greater risk of GC onset or peptic as well as duodenal ulcer disease and depends on the duration of infection and a dose of *H. pylori* [222,223]. The synthesis of CagA and VacA is believed to be regulated by OipA expression [224]. OipA is more prevalently detected in the gastric biopsy specimens obtained from patients with gastric precancerous lesions than those with gastritis alone. OipA promotes the secretion of numerous pro-inflammatory cytokines such as IL-1, IL-6, IL-8, IL-11 IL-17, matrix metalloproteinase 1 (MMP-1), tumor necrosis factor α (TNF-α), or RANTES [225,226,227,228,229,230]. It was reported that OipA might regulate β-catenin levels [231]. OipA suppresses the maturation of the dendritic cells by downregulating CD40, CD86, and MHC-II expression on the cell surface, as well as the release of IL-10 [232]. The synergistic activity between the virulence factors was presented in *H. pylori* strains that co-express the *oipa* gene with *caga*, and *vaca* [233]. OipA is involved in the translocation of CagA into the host gastric epithelial cells; this finding might be an explanation of why CagA-positive *H. pylori* strains usually express high amounts of Oipa [234,235]. OipA significantly increases the miR-30b levels affecting the miR-30b/xCT pathway and a further decrease in glutamate levels that stimulate *H. pylori*-induced damage [236]. 

## 15. Duodenal Ulcer Promoting Gene A

Duodenal ulcer promoting gene A (DupA) is the *H. pylori* virulence factor where pathogenicity is associated with the induction of duodenal ulcers as well as the risk of gastritis; nevertheless, DupA expression is negatively correlated with the risk of GC [237,238]. Moreover, DupA is even considered to be a protective factor preventing gastric carcinogenesis [239]. Although, Takahashi et al. (2013) showed that long-type *dupa* strains might contribute to GC via previously induced gastritis [240]. Interestingly, particular DupA-positive *H. pylori* strains do not always increase the risk of duodenal ulcers [241]. There is no association between DupA expression and the presence of other virulence factors such as CagA or VacA. DupA does not trigger bacterial adherence to gastric epithelial cells as well as the delivery of toxins to the host cells; however, the *dupa* gene is considered to be involved in the formation of T4SS [242]. The prevalence of DupA expression in *H. pylori* strains is estimated at 45% and significantly differs depending on the geographic area and the investigated ethnic groups [243,244]. A complete *dupa* cluster enables a greater infiltration of the inflammatory cells and is generally associated with greater virulence compared to strains with an incomplete *dupa* cluster [245]. DupA stimulates urease and IL-8 secretion primarily in the antrum contributing to the induction of gastritis; stimulation of the pro-inflammatory cytokine release by DupA also increases the risk of duodenal ulcers [246,247]. Furthermore, DupA facilitates inflammation within the antral mucosa and decreases corpus atrophy [248]. Except for IL-8 secretion, DupA triggers IL-12 (IL-12p40 and IL12-p70) production and secretion from the monocytes [249,250]. Contrarily to other *H. pylori* virulence factors, DupA inhibits the proliferation and growth of GC cells, preventing further carcinogenesis primarily via the overactivation of the mitochondria-mediated apoptotic pathway as well as high tolerance of DupA-positive strains to the acidic gastric microenvironment [251]. Infection by the DupA-positive strains might decrease the severity of *H. pylori*-related gastrointestinal pathologies and provide better clinical outcomes of patients. Furthermore, DupA stimulates gastric acid secretion, which was demonstrated by the presence of higher serum gastrin levels in patients infected by DupA-positive strains [252]. DupA is also associated with a greater failure of *H. pylori* eradication therapies [253]. It is also considered to be a diagnostic marker of *H. pylori*-related gastrointestinal diseases; so far, the *dupa* gene without premature stop codons or the 112 bp region of *H. pylori* DupA specifically could act as potential candidates [254,255,256,257,258]. 

## 16. Adherence-Associated Lipoprotein A and B 

Adherence-associated lipoprotein A and B (AlpA/AlpB) are proteins involved in *H. pylori* adherence to the gastric epithelial cells and further stimulation of bacterial colonization [259,260,261]. AlpA/B-deficient *H. pylori* mutants show significantly decreased capability to bind to the gastric epithelial cells compared to strains that express high levels of AlpA/B [262]. AlpA/B neither present toxic activity itself nor cross-react with human tissue antigens [263]. AlpA/B can be isolated from different *H. pylori* strains independently of geographic localization. AlpA/B can also regulate the release of several cytokines and pro-inflammatory factors including IL-6 and IL-8 and might be involved in the activation of the ERK, c-Fos, and CREB pathways; some of the AlpA/B genotypes might affect JNK and NF-κB pathways as well [262]. It was demonstrated that AlpB is involved in the formation of the bacterial biofilm [264]. Even though AlpA/B might promote *H. pylori* adherence, the lack of AlpA/B expression results in the enhanced inflammatory responses and more severe inflammation [265]. 

## 17. LacdiNAc-Specific Adhesin

LacdiNAc-specific adhesin (LabA) is an adhesin that facilitates *H. pylori* binding to the gastric epithelial cells via the recognition of the LacdiNAc (N, N^1^-diacetyllactosdiamine/GalNAcb4GlcNAc, LDN) determinant [266,267]. LabA is so far poorly characterized and more research should be done in order to assess its involvement and relevance in *H. pylori*-mediated pathogenicity.

## 18. *Helicobacter pylori* Outer Membrane Protein Q 

*Helicobacter pylori* outer membrane protein Q (HopQ) is a virulence factor relevant in bacterial survival in the gastric acidic microenvironment as well as the adherence to gastric epithelial cells, colonization, and further progression of gastrointestinal ailments. Two major types of HopQ can be distinguished—type 1 and 2—where expression might influence GC onset and progression in *H. pylori*-infected individuals. Generally, HopQ type 1 is present in the majority of *H. pylori* strains, which also possess cagPAI [268]. The genotype diversity of HopQ is associated with different severity of *H. pylori*-induced gastrointestinal pathologies; type 1 HopQ is relatively more often found in CagA and s1-VacA-positive strains, increasing the risk of peptic ulcer disease [269,270]. Furthermore, HopQ type 1 facilitates the infiltration of pro-inflammatory cells and increases gastric mucosal atrophy [271]. The HopQ type 2 genotype is rather more prevalent in CagA-negative s2-VacA-positive strains [272]. HoQ expression depends on a particular geographic area and ethnic differences and was reported to be associated with different clinical outcomes of patients. HopQ type 2 is rather associated with non-ulcer dyspepsia (NUD) and mild severe gastritis, whereas the presence of both—type 1 and 2—alleles significantly facilitates the risk of NUD. Usually, HopQ type 2 strains are not associated with peptic ulcer disease. The synergy between HopQ and CagA co-expression is recognized as a facilitated activity of several pro-inflammatory pathways, including NF-κB or those associated with mitogen-activated protein kinases (MAP kinases) via T4SS since HopQ significantly facilitates T4SS activity [273]. The interaction with T4SS and CagA translocation and/or phosphorylation induced by HopQ is stimulated by HopQ binding to host the carcinoembryonic antigen-related cell adhesion molecules (CEACAM) proteins (primarily CEACAM1, CEACAM3, CEACAM5, and CEACAM6) expressed on gastric epithelial cells promoting NF-κB activation [274,275,276,277,278,279,280]. This, as a consequence, might induce the development of gastric ulcers and facilitate gastric carcinogenesis. HopQ-induced CagA translocation is facilitated by HP0231, a major *H. pylori* thioloxidoreductase [281]. HopQ–CEACAM interactions are also crucial for *H. pylori* survival in neutrophils [282]. Furthermore, HopQ binding to CEACAM1 inhibits natural killer and T cell functions; such interactions are observed during the early stages of tumorigenesis [283]. HopQ-deficient strains present decreased capability to induce pro-inflammatory responses in the host cells as well as fail in CagA translocation [284]. HopQ–CEACAM interactions are considered to be potential therapeutic targets in the prevention and treatment of *H. pylori*-related gastrointestinal pathologies. 

## 19. *Helicobacter pylori* Outer Membrane Protein Z 

*Helicobacter pylori* outer membrane protein Z (HopZ) is another member of the OMPs family where pathogenicity is not clearly described yet compared to other OMPs. HopZ is divided into two variants—HopZ1 and HopZ2 [285]. HopZ facilitates bacterial adherence to gastric epithelial cells and stimulates *H. pylori* colonization in gastric mucosa; however, the exact site of HopZ binding is still unknown. HopZ pathogenicity is considered to be the most essential during the early stages of *H. pylori* infection; HopZ disturbs gastric acid secretion and is involved in the transient hypochlorhydria [286].

## 20. Induced by Contact with Epithelium Gene A

Induced by contact with epithelium gene A (IceA) is a quite recently discovered virulence factor of *H. pylori* that is still poorly described. IceA levels significantly increase during *H. pylori* binding with the gastric epithelial cells. There are two major allelic variants of IceA—IceA1 and IceA2. Since *H. pylori* possess only one *iceA* locus, IceA1 or IceA can be expressed and the presence of both indicates infection by several different *H. pylori* strains. Some researchers demonstrated that there is no relationship between IceA expression and the presence of other virulence factors such as CagA or VacA; however, these data are quite contradictory among authors, thus more research is needed in this field [287,288,289]. It was shown that the expression of IceA, similarly to other major virulence factors such as CagA or VacA, leads to poorer clinical outcome of the infected patients. Furthermore, IceA status significantly differs depending on particular geographic areas [290]. The expression of IceA increases during *H. pylori* binding with the gastric epithelial cells and might be associated with the development of peptic ulcer disease [287,290,291]. IceA1 strains are more prone to induce oxidative DNA damage which can be considered as a marker of GC progression or the risk factor related to the development of this malignancy [292,293]. Contrarily, IceA2 expression is more often associated with non-peptic ulcer dyspepsia [294]. IceA1-positive strains are capable of producing significant amounts of pro-inflammatory IL-8, which was not observed in cases of IceA1-negative strains [295,296]. Even though IceA1 expression is more prevalent among *H. pylori* strains, IceA2 presence is associated with greater granulocytic and lymphocytic infiltration, as well as atrophic gastritis [297]. Both allelic variants might facilitate the development of gastritis, gastric ulcers, functional dyspepsia, acute antral inflammation, as well as GC. Moreover, infection by the IceA-2-positive strains stimulates pro-inflammatory IL-1 polymorphisms, which is further associated with a greater risk of the onset and progression of the precancerous lesions in the gastric mucosa. Strains that express IceA, CagA, and VacA s1/m1 at the same time are likely to increase the severity of gastric inflammation [298]. IceA is considered to be a potential biomarker of the severity of gastrointestinal pathologies induced by *H. pylori*.

## 21. Cholesteryl α-Glucosyltransferase

Cholesteryl α-glucosyltransferase (αCgT) is a quite poorly characterized enzyme of *H. pylori* that is involved in the formation of cholesteryl α-glucoside (αCGL) by adding α-glucosyl to the cholesterol of the host cellular membranes. *H. pylori* does not synthesize cholesterol on its own but extracts it from the plasma membranes of the host cells; αCgT is involved in the subsequent glycosylation of the cholesterol. αCgT is produced by *H. pylori* in an inactive form which becomes activated after binding to the host cell membrane [299]. The absence of the *αCgT* gene is associated with significant dysregulations of the cholesterol uptake by the bacterium [300]. Glucosylation promoted by αCgT prevents *H. pylori* from the phagocytosis and immune responses induced by bacterial infection [301]. Furthermore, cholesteryl glucosides (CGs) are involved in the regulation of the responses from the CD4^+^ T-cells, as well as the IL-4 and IFN-γ pathways [302]. Increased αCgT expression inhibits IFNG-JAK/STAT1, as well as the IL-6 and IL-22 signaling pathways, resulting in an enhanced bacterial escape from the host inflammatory responses and further progression of gastric carcinogenesis [303]. αCgT is essential for the CagA translocation and stimulates the excessive secretion of IL-8 [304]. The levels of αCgT measured from the gastric tissue samples obtained from the *H. pylori*-infected patients are positively correlated with the atrophy score of the gastric tissues [305]. Thus, it was proposed that greater levels of αCgT might induce stronger immune responses that eventually lead to more severe *H. pylori*-induced inflammation, especially during the early stages of infection. Furthermore, αCgT also seems to be crucial for proper bacterial growth [306]. As an example, Lee et al. (2006) showed that *H. pylori* strains deficient in αCgT result in inhibited growth or even enhanced bacterial lethality [307]. αCgT-positive strains are more likely to survive in the macrophages due to the dysregulated autophagy processes; moreover, αCgT promotes bacterial survival in the host cells by interrupting autophagosome–lysosome fusion [308,309]. *H. pylori* strains that lack CGs show greater permeability of the cellular walls, decreasing bacterial pathogenic potential [310]. At the same time, the presence of GCs also seems to be crucial in bacterial antibiotic resistance. Therefore, αCgT might constitute a potential molecular therapeutic target for GC, peptic ulcers, or MALT lymphoma induced by the *H. pylori* infection. 

## 22. γ-Glutamyl-Transpeptidase

γ-glutamyl-transpeptidase (GGT) is one of the virulent enzymes of *H. pylori* that stimulates the conversion of glutamine and glutathione into glutamate and ammonia as well as glutamate and cyteinylglycine, respectively. The hydrolysis of the glutamine facilitates the vacuolation process by VacA [311]. GGT promotes the release of ROS (particularly H_2_O_2_), inhibition of cellular proliferation, as well as progression of apoptosis and necrosis of the gastric epithelial cells via the excessive secretion of IL-8 (in an NFκB-dependent manner), IL-10, cyclooxygenase-2 (COX-2), inducible iNOS, and epidermal growth factor-related peptides at the same time, resulting in dysregulated cell cycle processes [312,313,314,315]. Furthermore, GGT-induced apoptosis is stimulated in a mitochondrial-dependent manner via the release of cytochrome *c* and further overactivation of the caspase pathway (caspase-3 and -9) [316]. Park et al. (2014) demonstrated that GGT might increase Ca^2+^ levels through the activation of the phospholipase C-inositol 1,4,5-trisphosphate (PLC–IP3) pathway, eventually enhancing apoptotic processes [317]. Major dysregulations are primarily due to the GGT-dependent cell cycle arrest in the G1 phase with the downregulation of cyclin A and E, Cdk 4 and 6, and the upregulation of p21 with p27 [318,319]. Apoptotic processes and enhanced inflammation were also observed in the case of human biliary cells infected by GGT-positive strains, which indicates that *H. pylori* GGT might also contribute to the progression of cholangiocarcinoma [320].

GGT stimulates bacterial colonization and persistence in the gastric microenvironment via regulating immune tolerance by inhibiting CD4-positive T-cell proliferation, facilitating CD8^+^ cells infiltration into the gastric microenvironment, and preventing differentiation of the dendritic cells, triggering alterations in their phenotype into the tolerogenic one [321,322,323,324]. GGT overload downregulates major pathways crucial for proper T-cell functioning such as cMyc or interferon regulatory factor 4 (IRF4) and disrupts T-cell proliferation by inhibiting CD25 expression and IL-2 release [325]. GGT-negative *H. pylori* mutants fail in colonizing the gastric epithelium efficiently [326,327]. Furthermore, GGT is crucial for proper bacterial growth as GGT inhibition results in growth retardation [328]. GGT acts synergistically with other virulence factors such as VacA, inhibiting CD25 and CD69 expression and decreasing IL-2, IL-4, IL-10, and IFN-γ levels; IFN-γ levels are highly dependent on GGT activity [302]. Impaired and dysregulated immune responses stimulated by GGT might further contribute to the progression of gastric carcinogenesis. Since GGT is involved in DNA damage as well as cellular hyperproliferation, these processes might significantly contribute to the progression of gastric epithelial cells into the malignant phenotype [329]. GGT-positive *H. pylori* strains also show an ability to reduce cell viability and promote the loss of survivin [330]. Apart from gastric carcinogenesis, GGT might contribute to the onset and progression of peptic ulcer disease as well [331].

## 23. Neutrophil-Activating Protein 

Neutrophil-activating protein (NAP) is an *H. pylori* virulence factor that stimulates neutrophil adherence to the gastric epithelial cells, promoting the production of ROS as well as myeloperoxidase primarily during the stationary phases of infection. NAP activates the neutrophils and mast cells as well as promotes monocytes migration. Neutrophils are primarily stimulated due to the overactivation of the ERK and p38-MAPK pathways [332]. NAP presents pro-inflammatory activities and is highly involved in the progression of inflammation and tissue damage during *H. pylori* infection. NAP shows structural similarity to ferritin and belongs to the DNA-protecting protein under severe conditions (Dps) family. It presents ferroxidase activity however, without directly binding to it; due to the presence of the ferroxidase center, NAP incorporates Fe2+ ions that are further oxidized to Fe3+ and the whole process results in hydroxyl radicals that protect *H. pylori* DNA from damage [333]. Except for iron ions, NAP presents an ability to store other ions such as zinc or cadmium [334].

NAP stimulates the infiltration of monocytes and polymorphonuclear granulocytes as well as the surface expression of β2-integrins; furthermore, it stimulates the human blood mononuclear cells to secrete tissue factor (TF) and plasminogen activator inhibitor-2 (PAI-2), leading to the imbalance between coagulation and fibrinolysis [335]. Moreover, NAP induces the release of several chemokines such as interleukin-8 (IL-8), MIP-1α or CCL3, and MIP-1β (CCL4) from the neutrophils, tumor necrosis factor alpha (TNF-α), IL-6, and IL-8 by monocytes, along with the release of β-hexosaminidase and IL-6 by mast cells [336,337]. NAP prolongs the myeloid cells’ activation by protecting them from apoptosis by increasing the amounts of the anti-apoptotic proteins of the Bcl-2 family [338]. One of the most prominent NAP functions is the ability to stimulate the neutrophils activation, inducing a further release of IL-12 and IL-23 that facilitate Th-1 immune responses, with the excessive release of IFN-γ at the same time inhibiting Th-2 responses [335,339,340,341,342].

The release of the above-mentioned factors significantly increases the secretion of gastrin and pepsinogen and subsequent damage to the gastric mucosa. These alterations might contribute to the progression of chronic gastritis via the disrupted processes of fibrin removal and further impaired healing processes. Furthermore, NAP stimulates the mast cells to secrete β-hexosaminidase and IL-6, facilitating a pro-inflammatory cascade [343]. Its involvement in the maintenance of chronic inflammation is also observed as a NAP-induced Ag-specific T cells overactivation and intensification of the dendritic cells’ maturation and subsequent migration [344]. One of the hypotheses regarding NAP functions is its binding to the iron. However, its importance in inflammation is also presented by impairing the epithelial tight junctions and basal membranes within the gastric mucosa; it is also believed to stimulate the release of nutrients providing *H. pylori* growth during infection [345,346]. NAP also facilitates *H. pylori* survival by its contribution to DNA protection [347]. 

## 24. High Temperature Requirement A

High temperature requirement A (HtrA) is an *H. pylori* serine protease that targets E-cadherin on the gastric epithelial cells, leading to the cleavage of the E-cadherin extracellular domain and subsequent impairments in epithelial barrier functions [348,349,350]. HtrA primarily disturbs two types of junctions within the epithelium adherens junctions (E-cadherin) and tight junctions (occludin and claudin-8), as well as some of the extracellular matrix proteins, including aggrecan, proteoglycans, and fibronectin [351]. HtrA is localized in the *H. pylori* OMVs. Several studies reported that inhibition of *H. pylori* HtrA results in the blockage of *H. pylori* passage through the gastric epithelium as well as the reduced number of alterations within the gastric epithelial cells [352,353]. HtrA might increase *H. pylori* migration properties even up to 2.2-fold when compared to HtrA-negative species [354]. The transport of this virulence factor into the periplasm is provided by the presence of the signal peptide responsible for Sec-dependent transport through the inner membrane. Apart from *H. pylori* transmigration through the gastric epithelium, HtrA plays a role in CagA injection into the host cells by interacting with the α5β1 receptor [355]. HtrA expression is significantly increased in low pH and under stress conditions. The *htrA* gene is highly prevalent in numerous *H. pylori* strains regardless of the geographic areas. What is intriguing is that HtrA inhibition might be lethal for *H. pylori* strains, indicating that it constitutes a pivotal virulence factor of *H. pylori*; zinc and copper ions were presented to be effective inhibitors of the HtrA activity [356,357]. Moreover, excessive amounts of calcium ions seem to prevent the HtrA-related E-cadherin cleavage along with the additional stabilization of the junctions [358]. Furthermore, *H. pylori* strains that are deprived of HtrA are much more sensitive to particular conditions, including elevated temperature, osmotic shock, or treatment with puromycin [359,360]. Indeed, HtrA constitutes a crucial protective factor that prevents *H. pylori* from numerous stress conditions, enabling effective bacterial colonization within the gastric mucosa. HtrA is suspected to be one of the potential factors that might induce EMT and further tumorigenesis; however, so far, there is not sufficient data to provide an ultimate confirmation of this phenomenon. 

## 25. Heat Shock Proteins

Heat shock proteins (Hsps) constitute a family of proteins that act as molecular chaperones, being responsible for the maintenance of proper structural and functional properties of the cellular proteins. Except for the above-mentioned chaperoning role, Hsps also regulate apoptosis, autophagy, immunity, inflammation, carcinogenesis, as well as the protection against oxidative stress. There are three major Hsps expressed by *H. pylori—*HspB (Hsp60), HspA (Hsp10), and Hsp70—and their expression is regulated by two major repressors—HspR and HrcA. HspB affects the Nrf2/Keap1 pathway, inhibiting Nrf2 translocation, subsequent increased release of COX-2, IL-8, and metalloproteinases (MMP3 and MMP7), and enhanced inflammatory responses [361]. HspA is crucial for proper urease activation during *H. pylori* infection. HspA of *H. pylori* shares approximately 50% similarity to human homologues of Hsp60 [362]. HspA binds to the nickel ions as well as bismuth; thus, it might constitute a potential molecular target for potential anti-ulcer drugs [363]. HspA60-mediated inflammation within the gastric mucosa is due to its interactions with the TLR and further overactivation of the NF-κB, ERK, and MAP kinase (MAPK) signaling pathways with a subsequent IL-8 and TNF-α secretion from the monocytic cells [364,365,366,367]. Macrophages are stimulated by Hsp60 to release the IL-6 due to the overactivation of the NF-κB pathway as well as IL-10 and TGF-β that stimulate the proliferation of Treg cells promoting inflammation [368,369]. Furthermore, Hsp60 is involved in *H. pylori* adherence and further colonization within the gastric epithelium. The C-terminal domain of HspA was reported to play a role in nickel sequestration and detoxification and since nickel homeostasis is crucial for *H. pylori* colonization, this virulence factor might act as a potential molecular target for *H. pylori* eradication [370]. *H. pylori* motility is highly associated with the expression of Hsps inhibitors—HspR and HrcA [371]. It was demonstrated that HspA seropositivity increases with an individual’s age [372]. Hsp60 diffuses from the *H. pylori* into the gastric epithelial cells, leading to persistent bacterial infection, inflammation, and subsequent carcinogenesis via the enhancement of tumor cell migration and angiogenesis. Hsp60-mediated angiogenesis is primarily promoted by the CXCR2/PLCb2/Ca2+ signaling pathways in gastric epithelial cells [373]. Hsp60 might also constitute a potential predicting factor concerning *H. pylori* eradication on the gastric MALT lymphoma [374,375,376]. Furthermore, the cross-reactions of Hsps, e.g., between the human Hsp60 and bacterial HspB, are common and they significantly facilitate the onset of gastrointestinal diseases [362]. Apart from the Hsps that are expressed by *H. pylori*, bacterial colonization in the gastric mucosa significantly decreases the expression of host Hsps such as Hsp70 or Hsp90, and at the same time increases Hsp32 and Hsp27 levels, which additionally increases the susceptibility to further damage [377,378,379,380,381].

## 26. Conclusions

This review provides an insight into the virulence of *H. pylori* and its impact on the gastric microenvironment in the current state of knowledge. Colonization of different *H. pylori* strains in the same individual at the same time indicates that *H. pylori* is capable of inducing various genetic alterations and the expression of numerous virulence factors during its colonization period. Specific virulence factors are associated with the severity of symptoms and the clinical outcome of the infected patients. Several bacterial antigens such as BabA, SabA, OipA, AlpA, DupA, GGT, NAP, catalase, or Hsp60 are considered to be potential candidates for vaccines that might elicit both humoral and cellular immune responses during the infection. *H. pylori* elicits numerous adaptive mechanisms that enable effective bacterial adherence, colonization, and cellular alterations that provide the induction of further premalignant changes in the gastric microenvironment. Even though *H. pylori* virulence factors might act synergistically, some of them are crucial in bacterial colonization and thus chronic infection, whereas others might only act as additional triggers that only stimulate further responses. Therefore, more research regarding the *H. pylori* structure and virulence as well as their association with gastrointestinal diseases is needed. 

## Figures and Tables

**Table 1:** Characteristics of **Helicobacter pylori** virulence factors that facilitate carcinogenesis and protect the bacterium from the gastric microenvironment, enabling colonization and proliferation.

Virulence Factor	Function
Urease	Protects from the gastric acidity Facilitates bacterial colonization Stimulates bacterial nutrition Generates the proton motive force Modulates the host immune responses (facilitated apoptosis, chemotaxis of neutrophils and monocytes, altered opsonization, enhanced release of the pro-inflammatory cytokines) Stimulates platelet activation Stimulates angiogenesis
Flagellum	Enhances bacterial motility Stimulates chemotaxis Takes part in the biofilm formation Facilitates inflammation and immune evasion
Cytotoxin-associated gene A	Stimulates inflammatory responses Induces the release of IL-8 and IL-12 Enhances bacterial motility Activates RUNX3, ASPP2, CDX1, and fibroblasts Induces EMT Stimulates host cell growth and proliferation Reduces the activity of PDCD4, GSK-3, microRNA-134, Afadin protein, heat shock proteins Stimulates the induction of cancer stem cell-like properties
Vacuolating cytotoxin A	Involved in the formation of pores Promotes the autophagy pathways Forms the intracellular vacuoles and impaired autophagosomes Induces apoptosis and necrosis Inhibits the activity and proliferation of T and B cells Inhibits the IFN-β signaling inducing macrophage apoptosis Induces the release of IL-8 Differentiation of the regulatory T cells into effector T cells Prevents cellular elongation by inhibiting the Erk1/2 kinase pathways
Catalase	Induces mutagenesis Facilitates inflammation Protects **H. pylori** from complement-mediated killing Maintains bacterial survival at the cell surface of the phagocytes and in the macrophage phagosomes Protects **H. pylori** from phagocytosis
Superoxidase dismutase	Facilitates bacterial colonization Protects from ROS Inhibits the production of pro-inflammatory cytokines Stimulates the activation of the macrophages
Lewis antigens	Protects **H. pylori** from the host defense mechanisms Enhances bacterial survival Enhances the adhesive properties and further internalization
Arginase	Stimulates apoptosis Prevents bacterial killing Provides acid resistance in the gastric microenvironment Inhibits the proliferation of T cells Inhibits the production of NO Impairs the host immune responses Induces the apoptosis of macrophages Impairs Th1/Th17 differentiation
Phospholipases	Degradation of various lipids Damage the mucus layer Stimulate chronic inflammation Facilitate bacterial colonization and survival Activate the ERK1/2 signaling pathway
Lipopolysaccharide	Induces the host inflammatory responses by molecular mimicry Protects the bacterium from potentially toxic compounds Activates the TLR4, TLR2, PD-1, MMP-9, PLC/PKC/PI3K, JAK/STAT pathway, TNF-α, IFN-γ, IL-10, IL-12, IL-18, EPA-78, MCP-1, IL-8, IL-1β, and -4, -6, -7, -9 claudins Facilitates Th1 immune responses Activates neutrophils promoting oxidative stress reactions Induces monocyte inflammatory responses and monocyte transendothelial migration Disrupts the mucus secretion
Blood group antigen-binding adhesin	Enables bacterial adherence to gastric epithelial cells Stimulates the delivery of toxins (due to the increased T4SS activity) Stimulates the inflammatory responses (excessive IL-8 release, granulocyte infiltration)
Sialic acid-binding adhesin	Stimulates neutrophil activation and infiltration Facilitates bacterial colonization Induces the oxidative damage
Outer inflammatory protein A	Activates the apoptotic cascade Promotes the secretion of the pro-inflammatory cytokines such as IL-1, IL-6, IL-8, IL-11 IL-17, matrix metalloproteinase 1 (MMP-1), TNF-α, RANTES Regulates β-catenin levels Inhibits the maturation of the dendritic cells Takes part in CagA delivery into the host cells Increases microRNA-30b levels
Duodenal ulcer promoting gene A	Involved in the formation of T4SS Stimulates the infiltration of the inflammatory cells Facilitates urease and IL-8 secretion and IL-12 release from the monocytes Activates the mitochondria-mediated apoptotic pathways Facilitates bacterial tolerance in the acidic microenvironment
Adherence-associated lipoprotein A and B	Enables bacterial adherence to gastric epithelial cells Stimulates bacterial colonization Takes part in the formation of the biofilm Induces the release of pro-inflammatory factors (IL-6 and IL-8)
LacdiNAc-specific adhesin	Facilitates adherence to gastric epithelial cells
*Helicobacter pylori* outer membrane protein Q	Facilitates adherence to gastric epithelial cells Enables bacterial survival in the acidic gastric microenvironment Stimulates the infiltration of pro-inflammatory factors Facilitates T4SS activity Enables the survival of neutrophils Inhibits natural killer and T cells functions
*Helicobacter pylori* outer membrane protein Z	Facilitates the adherence to gastric epithelial cells Disturbs gastric acid secretion
Induced by contact with epithelium gene A	Induces oxidative DNA damage Stimulates the release of pro-inflammatory factors (IL-8, IL-1) Facilitates granulocytic and lymphocytic infiltrations
Cholesteryl α-glucosyltransferase	Prevents **H. pylori** from the phagocytosis and immune responses Regulates the responses from the CD4^+^ T-cells, IL-4, and IFN-γ pathways Stimulates the secretion of IL-8 Crucial for proper bacterial growth, survival, and antibiotic resistance Interrupts the autophagosome-lysosome fusion
γ-glutamyl-transpeptidase	Induces the release of ROS Inhibits cellular proliferation Facilitates apoptosis and necrosis Incudes the release of IL-8, IL-10, COX-2, inducible iNOS, caspase-3 and -9 Inhibits CD4+ T cell proliferation and stimulates CD8+ T cells infiltration Prevents the differentiation of the dendritic cells Stimulates DNA damage Reduces cell viability
Neutrophil-activating protein	Facilitates neutrophil adherence to gastric epithelial cells Produces ROS and myeloperoxidase Activates neutrophils and mast cells and the migration of the monocytes Stimulates the infiltration of monocytes and polymorphonuclear granulocytes Stimulates the release of IL-8, MIP-1α, MIP-1β, TNF-α, IL-6, β-hexosaminidase Impairs the epithelial tight junctions and basal membranes Facilitates **H. pylori** growth
High temperature requirement A	Impairs the functions of the epithelial barrier by disrupting adherens junctions, tight junctions, and extracellular matrix proteins Facilitates **H. pylori** migration properties Promotes CagA injection into the host cells Prevents **H. pylori** from stress conditions
Heat shock proteins	Maintains the proper structural and functional properties of the cellular proteins Protects from the oxidative stress Regulates apoptosis and autophagy Crucial for proper urease activation Induces the release of COX-2, IL-8, TNF-α, MMP3, and MMP7 Facilitates adherence to gastric epithelial cells Facilitates tumor cell migrations Enhances angiogenesis

## Abbreviations

αCGL	cholesteryl α-glucoside
αCgT	cholesteryl α-glucosyltransferase
AlpA	adherence-associated lipoprotein A
AlpB	adherence-associated lipoprotein B
AmiE and AmiF	aliphatic amidases—E and F
ASPP2	apoptosis-stimulating protein of p53 2
BabA	blood group antigen binding fadhesin
cagPAI	cag pathogenicity island
CEACAM	carcinoembryonic antigen-related cell adhesion molecules
Cu/Zn-SOD	copper-zinc superoxide dismutase
CXCR7	CXC chemokine receptor 7
Dps	DNA-protecting protein under severe conditions
DupA	duodenal ulcer promoting gene A
EC-SOD	extracellular matrix superoxide dismutase
EMT	epithelial-mesenchymal transition
GC	gastric cancer
GGT	γ-glutamyl-transpeptidase
GERD	gastroesophageal reflux disease
GSK-3	glycogen synthase kinase 3
*H. pylori*	Helicobacter pylori
HopQ	*Helicobacter pylori* outer membrane protein Q
HopZ	*Helicobacter pylori* outer membrane protein Z
Hsps	heat shock proteins
HtrA	high temperature requirement A
IBD	inflammatory bowel disease
IceA	induced by contact with epithelium gene A
iNOS	nitric oxide synthase
LabA	LacdiNAc-specific adhesin
(Le) antigens	Lewis antigens
LPS	lipopolysaccharide
LRP-1	lipoprotein receptor-related protein-1
MALT	mucosa-associated lymphoid tissue lymphoma
MAP	mitogen-activated protein
MAPK	mitogen-activated protein kinase
MD-2	myeloid differential protein-2
MHC	major histocompatibility complex
MMP-1	matrix metalloproteinase 1
Mn-SOD	manganese superoxide dismutase
Mox1	mitogen oxidase 1
NAP	neutrophil-activating protein
NUD	non-ulcer dyspepsia
OipA	outer inflammatory protein A
OMP	outer membrane protein
OMPLA	outer membrane phospholipase A
OMVs	outer membrane vesicles
PAI-2	plasminogen activator inhibitor-2
PD-1	programmed death-ligand 1
PDCD4	programmed cell death protein 4
PLA_1_	phospholipase A1
PLA_2_	phospholipase A2
PLC	phospholipase C
PLC–IP3	phospholipase C – inositol 1,4,5-trisphosphate
PLD	phospholipase D
ROS	reactive oxygen species
RPTPα	receptor-like protein tyrosine phosphatases β
RPTPβ	receptor-like protein tyrosine phosphatases α
RUNX3	run-related transcription factor 3
SabA	sialic acid–binding adhesin
SOD	superoxide dismutase
T4SS	type 4 secretion system
TF	tissue factor
TFF	trefoil factor family
TGF-β1	transforming growth factor-beta 1
TLR4	toll-like receptor 4
TNF-α	tumor necrosis factor alpha
VacA	vacuolating cytotoxin
Vn	vitronectin
WHO	World Health Organization
YAP	yes-associated protein
